# 294. Assessment of Disinfectants for Reducing *Candida auris* Burden on Needleless Connectors: an In Vitro Study

**DOI:** 10.1093/ofid/ofae631.084

**Published:** 2025-01-29

**Authors:** Truc Cecilia Tran, Max W Adelman, Shiva Murali, Adriana Jimenez, Cesar A Arias, Bhavarth S Shukla

**Affiliations:** Houston Methodist Research Institute, Houston, TX; Houston Methodist Hospital, Houston, Texas; Houston Methodist Research Institute and Weill Cornell Medical College, Houston, Texas; University of Miami Health System, Miami, Florida; Houston Methodist and Weill Cornell Medical College, Houston, TX; University of Miami, Miami, Florida

## Abstract

**Background:**

*Candida auris*, an emerging fungal pathogen, has triggered outbreaks in healthcare settings. It frequently colonizes patient skin, surfaces in their immediate clinical environment, and medical devices, including central venous catheters and connectors. Effective disinfection is vital for patient safety. However, many commonly used disinfectants may have limited efficacy against *C. auris* and their ability to clean contaminated connects remain uncertain. This study assesses the effectiveness of commonly used disinfectants in reducing *C. auris* contamination in needleless connectors.

**Figure:**
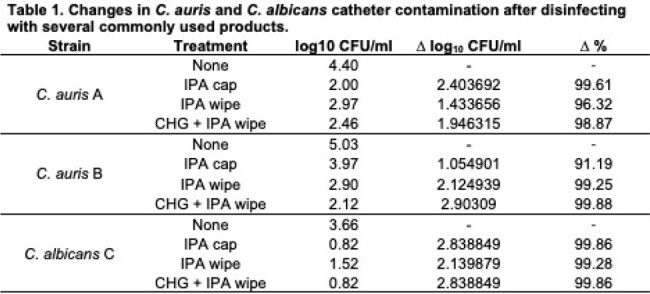

Changes in C. auris and C. albicans catheter contamination after disinfecting with several commonly used products.

**Methods:**

Needleless connectors of Arrowg+ard Blue Plus® Four-Lumen Catheters were contaminated with *C. auris* and *C. albicans* over a 24-hour period. Disinfection procedures involved using single-use disinfecting cap protectors containing 70% isopropyl alcohol (IPA), wipes with 70% IPA, and wipes containing 3.15% chlorhexidine gluconate plus 70% IPA (CHG + IPA). The disinfection process included mechanically rotating the wipes and caps in both clockwise and counterclockwise directions for 15 seconds, followed by allowing the connectors to dry for an additional 15 seconds. Treated and control connectors were then immersed in 1 ml of 0.9% NaCl solution, placed in an ultrasonic bath for 1 minute, and subsequently enumerated on Sabouraud dextrose agar.

**Results:**

Two strains of *C. auris* and one strain of *C. albicans* were used. Under untreated conditions, *C. auris* exhibited colonization ranging from 4.4 to 5.0 log_10_ CFU/ml. Disinfection procedures resulted in a reduction of *C. auris* fungal load by 1.43 to 2.90 log_10_ CFU/ml, indicating a 91.2% to 99.9% reduction in CFU/ml across all products. However, efficacy was lower with IPA alone (refer to Table 1). Against *C. albicans*, disinfecting products reduced the fungal load from 3.66 log_10_ CFU/ml to 0.82 to 1.52 log_10_ CFU/ml, representing >99.2% reduction in all conditions, with the most significant reduction observed with the IPA cap and CHG + IPA wipe.

**Conclusion:**

Our findings indicate that commonly used disinfectants have limited efficacy against *C. auris* colonization in needleless connectors compared to *C. abicans*. Further research is warranted to assess optimal methods and products for decontaminating devices from this emerging pathogen.

**Disclosures:**

**Cesar A. Arias, MD, MSc, PhD**, UpToDate, Inc.: Royalties

